# Comparative *in silico* analysis of miRNA targeting of NAC and MYB transcription factors associated with secondary cell wall regulation in rice and maize

**DOI:** 10.3389/fpls.2026.1887120

**Published:** 2026-09-03

**Authors:** Ayaz Belkozhayev, Nargiz Gizatullina, Gaukhargul Yelemessova, Madina Mussalimova, Ronagul Turganova, Bekzhan D. Kossalbayev, Aizhan Rakhmetullina, Anna Pyrkova, Anatoliy Ivashchenko, Gaukhar Toleutay

**Affiliations:** 1 Department of Chemical and Biochemical Engineering, Geology and Oil-Gas Business Institute Named After K. Turyssov, Satbayev University, Almaty, Kazakhstan; 2 Research Institute of Advanced Materials, Almaty, Kazakhstan; 3 L2A, INRAE-USC 0340, ENSAIA Department, Faculty of Science and Technology, University of Lorraine, Nancy, France; 4 Sustainability of Ecology and Bioresources, Al-Farabi Kazakh National University, Almaty, Kazakhstan; 5 Ecology Research Institute, Khoja Akhmet Yassawi International Kazakh Turkish University, Turkistan, Kazakhstan; 6 Institute of Biochemistry and Biophysics, Polish Academy of Sciences, Warsaw, Poland; 7 Department of Biotechnology, Al-Farabi Kazakh National University, Almaty, Kazakhstan; 8 Center for Bioinformatics and Nanomedicine, Almaty, Kazakhstan; 9 Department of Chemistry, University of Tennessee, Knoxville, TN, United States

**Keywords:** coding sequence (CDS), in silico analysis, miRNA, MYB transcription factors, NAC transcription factors, secondary cell wall regulation

## Abstract

Cellulose biosynthesis and secondary cell wall formation are regulated by hierarchical networks involving NAC and MYB transcription factors and their post-transcriptional regulation by miRNAs. Here, we performed a cross-species *in silico* analysis of the complete annotated NAC and MYB transcription factor gene sets of *O. sativa* subsp. *japonica* and *Z. mays* using MirTarget, with selected high-priority interactions independently assessed by psRNATarget. Predicted miRNA-binding sites (BSs) were identified in 138 of 170 rice NAC genes (81.2%), 110 of 130 rice MYB genes (84.6%), 87 of 189 maize NAC genes (46.0%), and 117 of 203 maize MYB genes (57.6%), corresponding to 591, 480, 331, and 487 predicted sites, respectively. Members of the miR164 family predominated among NAC candidates in both species, whereas MYB transcripts showed greater miRNA diversity. The analysis recovered the established miR164–NAC and miR159–MYB modules as positive benchmarks. In contrast, miR5075-3p–LOC_Os04g38740.1 emerged as a leading novel transcript-specific candidate, with concordant computational support from MirTarget and psRNATarget. These findings define comparative patterns of predicted miRNA targeting rather than experimentally validated regulatory interactions and provide prioritized hypotheses for direct functional testing.

## 1 Introduction

Plant cell walls are complex and highly organized structures that play a fundamental role in plant growth, mechanical support, and environmental adaptation (Ezquer et al., 2020). Cellulose, the most abundant biopolymer on Earth, constitutes the primary load-bearing component of both primary and secondary cell walls (SCW) and is therefore a key determinant of biomass accumulation and structural integrity in plants (Li et al., 2014; Belkozhayev et al., 2025b). Beyond its biological significance, cellulose represents a major renewable resource for agriculture, bioenergy, and the development of sustainable, bio-based materials. Understanding the molecular mechanisms that regulate cellulose biosynthesis is thus essential for improving plant biomass quality and for advancing environmentally friendly applications derived from plant resources (Turganova et al., 2025; Kossalbayev et al., 2025). Cellulose biosynthesis is orchestrated by cellulose synthase (CesA) complexes and coordinated networks of transcription factors that regulate genes involved in SCW formation (Lei et al., 2012). Among these regulators, NAC and MYB transcription factor families play central and conserved roles (Kumar et al., 2021; Ma et al., 2025). NAC transcription factors are widely recognized as master regulators of SCW biosynthesis (Golfier et al., 2017), controlling downstream MYB genes and structural genes involved in cellulose, hemicellulose, and lignin synthesis (Yu et al., 2023; Wang et al., 2011). MYB transcription factors, in turn, function as key modulators that fine-tune the expression of cell wall biosynthetic genes and contribute to tissue-specific and developmental regulation of secondary wall deposition (Imran et al., 2025). The coordinated activity of NAC and MYB transcription factors forms hierarchical regulatory networks that are essential for proper cell wall formation in cereals such as *Oryza sativa* (*O. sativa*) and *Zea mays* (*Z. mays*) (Zhao et al., 2025; Fan et al., 2014; Belkozhayev et al., 2025a). Rice cultivation in temperate continental and irrigated agricultural systems is predominantly based on varieties belonging to *O. sativa* subsp. *japonica*. Therefore, *O. sativa* subsp. *japonica* was selected as the primary subspecies for this study to ensure biological relevance and practical applicability. In addition, the *japonica* genome is characterized by high-quality annotation and well-curated miRNA and transcription factor datasets (Wang et al., 2021), providing a robust framework for systematic *in silico* analysis of miRNAs and mRNA interactions (Wen et al., 2016; Rakhmetullina et al., 2023). Beyond transcriptional control, post-transcriptional regulation has emerged as a critical layer governing gene expression in plants (Rakhmetullina et al., 2023). MicroRNAs (miRNAs), a class of small non-coding RNAs typically 20–24 nucleotides in length, regulate gene expression by guiding sequence-specific interactions with target mRNAs, leading to mRNA cleavage or translational repression (Dong et al., 2022; Zhakypbek et al., 2025). In plants, miRNA–mediated regulation is characterized by a high degree of complementarity between miRNAs and their targets, enabling precise and efficient control of gene expression (Zhakypbek et al., 2025; Guimaraes et al., 2025). Increasing evidence indicates that miRNAs play important roles in cell wall biosynthesis, stress responses, and developmental processes by targeting transcription factors and other regulatory genes (Asakura et al., 2023; Dong et al., 2022; Samad et al., 2017; Farooq et al., 2025). Several plant miRNA families have been implicated in the regulation of NAC and MYB transcription factors (Samad et al., 2017; Teng et al., 2023). However, most previous studies have focused on individual miRNA–gene pairs or specific developmental contexts, while systematic cross-species comparisons of miRNA targeting across complete NAC and MYB transcription factor families remain limited. Systematic comparative analyses of miRNA binding patterns, thermodynamic properties, and binding site (BS) localization within NAC and MYB genes across major cereal crops remain limited. Computational (*in silico*) approaches provide a powerful framework for large-scale prediction and characterization of miRNA–mRNA interactions. By integrating sequence complementarity, binding free energy, and positional information, in silico analyses enable the identification and prioritization of candidate miRNA BSs and facilitate the exploration of complex regulatory networks that are difficult to resolve experimentally at genome scale (Rakhmetullina et al., 2020; Chaudhary et al., 2022). Such analyses are especially valuable for crops with well-annotated genomes, including rice and maize, where extensive miRNA and transcription factor datasets are available. In this study, we performed a systematic cross-species *in silico* analysis of predicted miRNA targeting across the complete annotated NAC and MYB transcription factor gene sets of *O. sativa* and *Z. mays*. Using the MirTarget program, we systematically predicted miRNA BSs within NAC and MYB mRNAs, evaluated their thermodynamic stability, relative binding efficiency, and localization within coding sequences, and compared regulatory patterns between the two transcription factor families and plant species. By focusing on thermodynamically favorable candidate interactions, this study aimed to identify potentially conserved and species-associated patterns of miRNA targeting within NAC and MYB transcription factor families, which represent important regulatory components of secondary cell wall formation. The analysis was not restricted to a limited set of preselected candidate genes. Instead, the complete annotated NAC and MYB transcription factor gene sets available in PlantTFDB were examined for both species. This focused design was selected because NAC and MYB transcription factors constitute a central hierarchical regulatory module controlling secondary cell wall formation. Structural and regulatory genes representing the complete cellulose, hemicellulose, and lignin biosynthetic pathways were outside the predefined scope of the present study.

## 2 Materials and methods

Mature plant miRNA sequences for *O. sativa* subsp. *japonica* and *Z. mays* were retrieved from the miRBase database (https://mirbase.org/, accessed on 2 February 2026). A total of 738 rice miRNAs and 325 maize miRNAs were included in the analysis. Because miRBase entries may differ in annotation confidence and experimental support, database inclusion was not considered equivalent to experimental validation of miRNA function.

mRNAs of NAC and MYB transcription factor genes were obtained from the Plant Transcription Factor Database (PlantTFDB; https://planttfdb.gao-lab.org/, accessed on 2 February 2026).

For *O. sativa*, 300 mRNA sequences were analyzed, comprising 170 NAC and 130 MYB transcription factor genes. For *Z. mays*, 392 mRNA sequences were selected, including 189 NAC and 203 MYB transcription factor genes. Thus, the analysis included 692 NAC and MYB transcripts in total and was not limited to a small set of individually selected candidate genes. The complete annotated NAC and MYB transcription factor gene sets available in PlantTFDB were included for each species. All genes were subjected to the same sequence-processing, target-prediction, and thermodynamic-filtering procedures. Complete prediction datasets, including transcript identifiers, miRNA names, binding-site positions, interaction lengths, ΔG values, and ΔG/ΔGm ratios, are provided in **Supplementary Tables 1**-**S4**. **Supplementary Table 1**-**S4** collectively constitute a structured, machine-readable database of all predicted miRNA–mRNA interactions generated in this study and are provided in Excel format to facilitate data access, filtering, and reuse.

Only CDS regions were included in the present analysis to ensure consistent sequence availability and annotation comparability across all NAC and MYB transcripts from rice and maize. Complete and uniformly curated 5′UTR and 3′UTR sequences were not available for all transcript isoforms in the selected datasets, and their inclusion could therefore have resulted in uneven transcript coverage and annotation-related bias in the cross-species comparison. Nevertheless, plant miRNA BSs may occur in the CDS as well as in the 5′UTR and 3′UTR. Therefore, the CDS-only design may have excluded biologically relevant miRNA BSs located in untranslated regions and represents a limitation of the present study.

Publicly available gene expression data for *O. sativa* NAC transcription factor genes were retrieved from the Expression Atlas module integrated within the Plant Transcription Factor Database (PlantTFDB; https://planttfdb.gao-lab.org/, accessed on 10 February 2026). Transcript abundance values reported as transcripts per million (TPM) were collected across multiple vegetative and reproductive tissues, including leaf, leaf collar, leaf sheath, shoot, pre-flowering panicle, and post-flowering panicle. Heatmap visualization of tissue-specific expression profiles was generated using TBtools software (Chen et al., 2023), which enables graphical representation and hierarchical clustering of gene expression datasets. The expression analysis was used as an exploratory assessment to confirm that selected predicted NAC target transcripts were expressed across the analyzed rice tissues. Comparable expression analyses were not performed for rice MYB or maize NAC and MYB genes in the present study. In addition, the available tissue-level mRNA expression profiles were not matched to miRNA abundance data generated from the same biological samples; therefore, formal miRNA–target expression correlation analysis was not performed.

**Figure 1:**
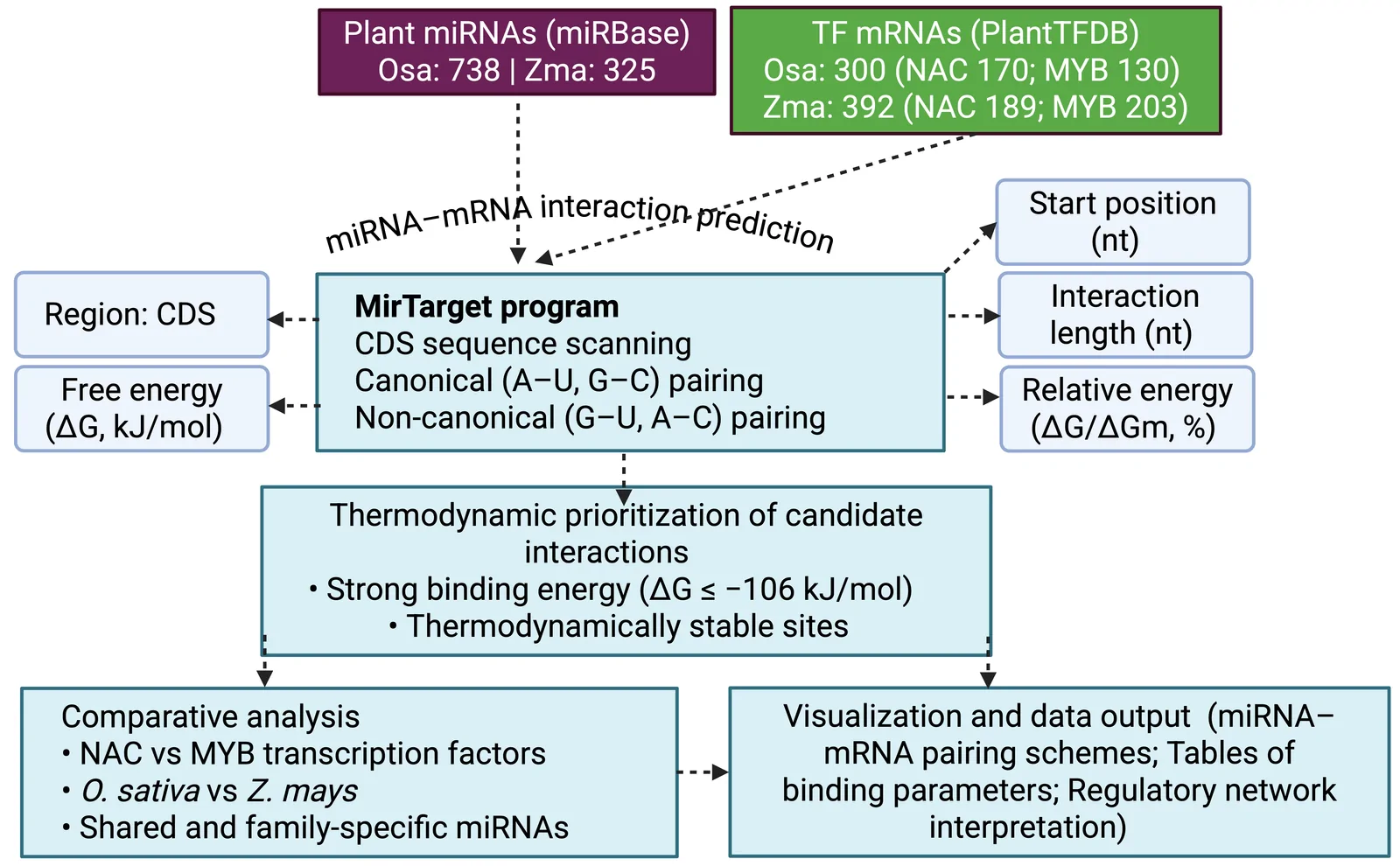
Workflow of the *in silico* analysis used to identify miRNA–mRNA interactions between plant miRNAs and NAC and MYB transcription factor genes in *O. sativa* subsp. *japonica* and *Z. mays*. Created with BioRender.com (License No. WD29C4YJUE).

Prediction of miRNA target genes was performed using the MirTarget program, which identifies miRNA and mRNA interactions based on sequence complementarity and thermodynamic stability (Ivashchenko et al., 2016b), (**Figure 1**). The MirTarget algorithm is capable of evaluating potential miRNA BSs across complete mRNA sequences, including the 5′UTR, CDS, and 3′UTR. In the present study, however, only CDS sequences were supplied to the program; therefore, all reported BSs were necessarily located within coding regions. The program calculated the free binding energy (ΔG, kJ/mol), relative binding efficiency (ΔG/ΔGm, %), interaction length, and binding-site position within the analyzed CDS sequences (Ivashchenko et al., 2016a; Ivashchenko et al., 2016b).

The maximum binding free energy (ΔGm) corresponds to the theoretical free energy of miRNA binding to a fully complementary nucleotide sequence. The ratio ΔG/ΔGm (%) was used as a comparative metric to assess the relative strength and stability of miRNA–mRNA interactions, with higher values indicating stronger and more energetically favorable binding. A distinctive feature of the MirTarget program is its ability to account for non-canonical nucleotide interactions in miRNA–mRNA duplex formation. In addition to conventional Watson–Crick base pairs (A–U and G–C), the algorithm considers G–U and A–C interactions, which are formed via a single hydrogen bond. These non-canonical pairs are treated as structurally compatible with canonical base pairs, allowing a more comprehensive and realistic assessment of miRNA binding potential in plant mRNAs (Friedman and Honig, 1995; Garg and Heinemann, 2018).

A ΔG threshold of ≤ −106 kJ/mol was applied as a stringent operational filtering criterion to prioritize the most thermodynamically favorable predicted miRNA–mRNA duplexes for detailed comparative analysis. This cut-off enabled the application of a uniform selection criterion across the rice and maize datasets and reduced the number of weaker candidate interactions included in the detailed analysis. However, ΔG ≤ −106 kJ/mol should not be interpreted as a universally validated biological threshold for miRNA targeting in rice or maize. Interactions with less negative ΔG values may also be biologically functional, whereas interactions meeting this criterion still require experimental validation.

MirTarget predicts miRNA BSs located within the 5′UTR, CDS, and 3′UTR of mRNAs. For each predicted BS, the program determines: (i) the nucleotide position of the BS start; (ii) its localization within mRNA regions; (iii) the free energy of miRNA–mRNA interaction (ΔG), and (iv) the nucleotide pairing scheme between miRNA and mRNA (Ivashchenko et al., 2016b; Belkozhayev et al., 2022; Belkozhayev et al., 2024).

To descriptively visualize nucleotide patterns among selected predicted miR164-compatible sites (ΔG ≤ −106 kJ/mol), sequence logo analysis was performed using the WebLogo 3 online server (https://weblogo.threeplusone.com/create.cgi, accessed on 14 February 2026). Aligned 21-nt predicted binding regions within the analyzed CDS sequences were used as input. The resulting logos were used only as descriptive visualizations of nucleotide frequencies and were not considered independent evidence of target validity or evolutionary conservation.

Candidate miRNA–mRNA interactions were further prioritized for future functional characterization using an integrative qualitative framework. The absolute ΔG value was not used as the sole ranking criterion because interaction length and the theoretical maximum binding energy differ among miRNAs. Candidate selection therefore considered the combined evidence from: (i) a ΔG value meeting the operational threshold of ≤ −106 kJ/mol; (ii) a high ΔG/ΔGm ratio; (iii) extensive or near-complete sequence complementarity across the predicted binding region; (iv) recurrent targeting patterns, including the occurrence of the same miRNA in multiple NAC or MYB transcripts or in both transcription factor families; and (v) the availability or absence of previous experimental evidence for the specific interaction. Publicly available expression data were used only as additional biological context for *O. sativa* NAC candidates and were not applied as a universal ranking criterion because equivalent expression datasets were not analyzed for all gene groups.

When multiple predicted BSs occurred within the same transcript or partially overlapped, the site with the more negative ΔG value was retained as the representative site-level interaction. This site-level selection rule was distinct from the integrative gene-level prioritization described above, which also considered ΔG/ΔGm, sequence complementarity, targeting pattern, biological context, and the availability of previous experimental evidence. The MirTarget algorithm has been applied in previous studies to predict candidate miRNA BSs across different species; however, the biological relevance of individual predicted interactions requires independent experimental validation (Ivashchenko et al., 2016a; Ivashchenko et al., 2016b; Belkozhayev et al., 2022; Belkozhayev et al., 2024).

All miRNA–mRNA interactions reported in this study should be interpreted as computational predictions. Favorable thermodynamic parameters and extensive sequence complementarity were used to prioritize candidate interactions but do not independently demonstrate transcript cleavage, translational repression, or functional regulation *in vivo*.

To provide independent computational support for selected high-priority predictions, two prioritized *O. sativa* miRNA–MYB interactions, osa-miR5075-3p–LOC_Os04g38740.1 and osa-miR159c-3p–LOC_Os05g41166.1, were additionally evaluated using psRNATarget (Dai et al., 2018). The same mature miRNA and CDS sequences used in the primary MirTarget analysis were submitted to psRNATarget. The analysis was performed using Schema V2 (2017 release) with the default scoring parameters and a maximum expectation value of 5.0; target accessibility calculation was not enabled. Candidate interactions recovered by both MirTarget and psRNATarget were considered concordantly supported computational predictions. This independent cross-validation was restricted to these two prioritized interactions and was not intended as a genome-wide validation of the complete prediction dataset.

To systematically assess the experimental support available for selected key miRNA–target interactions, evidence reported in previous studies was classified according to validation type, including degradome/PARE evidence, RLM-5′ RACE or 5′-RACE cleavage validation, reporter assays, genetic or functional evidence, and expression-based evidence. Evidence was considered target-specific only when it referred to the same miRNA–transcript pair analyzed in the present study. Evidence concerning the same miRNA family but a different target transcript was considered supportive of the broader regulatory module rather than direct validation of the specific candidate interaction.

No custom computational pipeline or newly developed source code was used in this study; therefore, no separate GitHub repository was created. All analytical steps, software tools, input databases, filtering criteria, and parameters are described in the Materials and Methods section and **Figure 1**.

For quantitative cross-species comparisons, the proportions of NAC and MYB genes containing at least one predicted miRNA BS were compared between *O. sativa* and *Z. mays* using two-sided Fisher’s exact tests. Effect sizes were expressed as odds ratios (ORs) together with absolute differences in targeted-gene proportions, and exact P values were reported. Statistical testing was restricted to gene-level targeting proportions. Individual predicted BSs were not treated as independent biological observations because multiple sites may occur within the same transcript and related miRNAs may generate correlated predictions. Consequently, site-level ΔG and ΔG/ΔGm values were summarized descriptively rather than subjected to inferential statistical testing. Statistical results were therefore interpreted as quantitative differences between the computational prediction datasets rather than as evidence of species-level biological regulation.

To account for differences in the numbers of analyzed and targeted transcripts among species and transcription factor families, predicted binding-site abundance was additionally normalized by gene number. Two complementary metrics were calculated: the total number of predicted BSs divided by the total number of genes analyzed (BSs per analyzed gene) and the total number of predicted BSs divided by the number of genes containing at least one predicted site (BSs per targeted gene). These normalized metrics were used together with, rather than replaced by, the absolute binding-site counts to describe the computational prediction datasets.

## 3 Results

For the analysis of predicted miRNA targeting of NAC and MYB transcription factor genes, the complete annotated NAC and MYB gene sets of O. sativa subsp. japonica and Z. mays were examined. The MirTarget program was used to predict miRNA–mRNA interactions based on sequence complementarity and binding free energy. In total, 300 rice transcripts, comprising 170 NAC and 130 MYB genes, were analyzed against 738 rice miRNAs, whereas 392 maize transcripts, comprising 189 NAC and 203 MYB genes, were analyzed against 325 maize miRNAs. Thus, the dataset represented the complete annotated NAC and MYB transcription factor families available for the two species rather than a limited selection of individual candidate genes.

**Figure 2:**
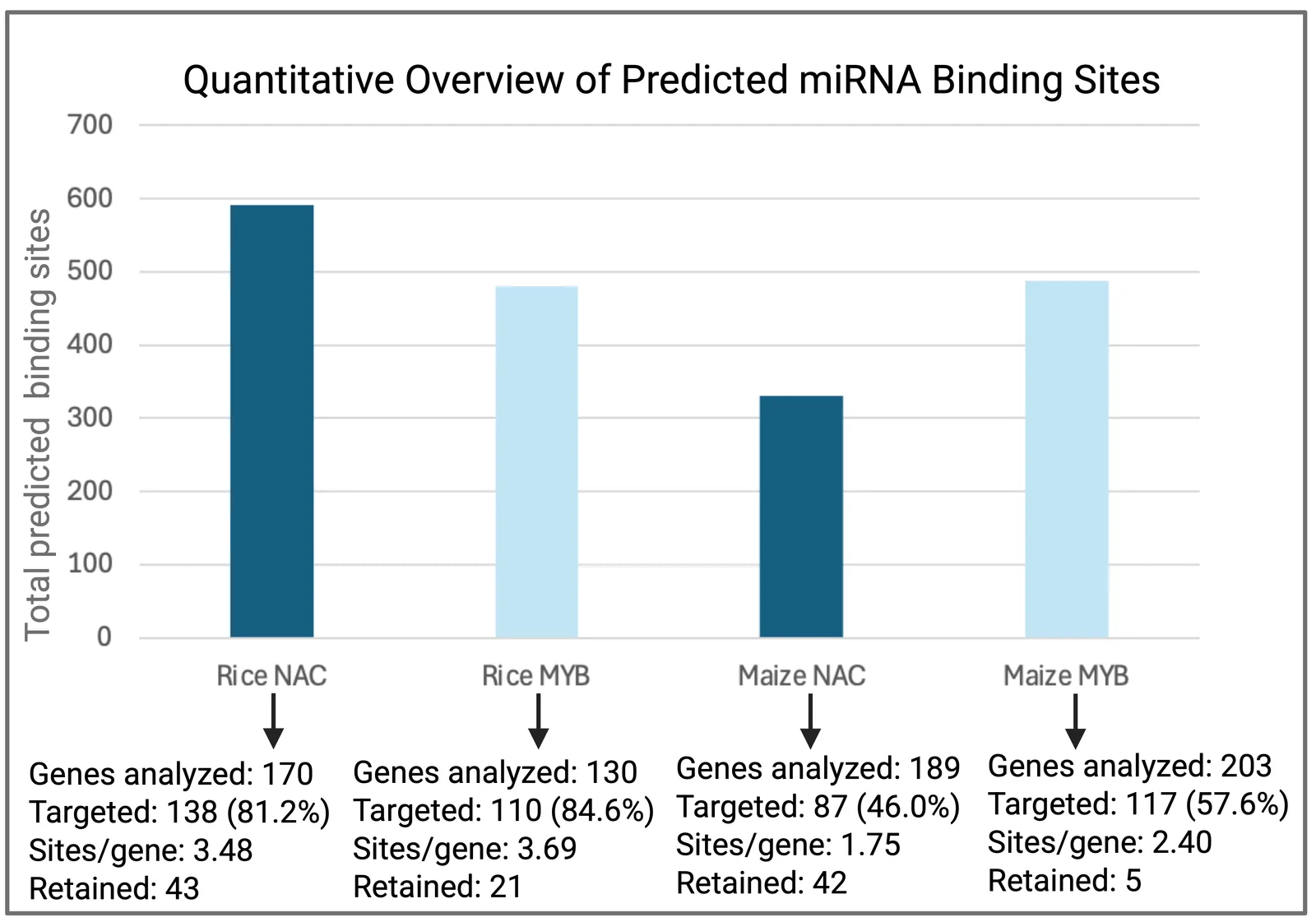
Quantitative overview of predicted miRNA targeting in NAC and MYB transcription factor gene families of *O. sativa* and *Z. mays*. Bars indicate the total number of predicted miRNA BSs. Additional annotations show the number of genes analyzed, the number and percentage of genes containing at least one predicted site, the number of BSs per analyzed gene, and the number of candidate BSs retained after application of the stringent operational criterion (ΔG ≤ −106 kJ/mol).

To provide a concise quantitative overview, the complete prediction datasets were compared across species and transcription factor families (**Figure 2**; **Table 1**). In *O. sativa*, predicted BSs were identified in 138 of 170 NAC genes (81.2%) and 110 of 130 MYB genes (84.6%), resulting in 591 and 480 BSs, respectively. In *Z. mays*, 87 of 189 NAC genes (46.0%) and 117 of 203 MYB genes (57.6%) contained predicted BSs, yielding 331 and 487 sites, respectively.

**Table 1:** Comparative summary of predicted miRNA targeting patterns in NAC and MYB transcription factor genes of *O. sativa* and *Z. mays*.

Species	TF family	Genes analyzed	Targeted genes	Predicted miRNAs	Total BSs	BS per analyzed gene	BS per targeted gene	Principal candidate miRNAs	Main pattern
*O. sativa*	NAC	170	138 (81.2%)	181	591	3.48	4.28	miR164 family, miR5075-3p, miR2102-3p/5p	Predominance of miR164 and recurrent multiple site targeting
*O. sativa*	MYB	130	110 (84.6%)	164	480	3.69	4.36	miR2102-5p, miR5075-3p; miR159c-3p as a gene-specific candidate	Broader miRNA diversity, including multi-target and gene-specific patterns
*Z. mays*	NAC	189	87 (46.0%)	106	331	1.75	3.80	miR164 family, miR167d-3p	Strong predominance of the miR164 family
*Z. mays*	MYB	203	117 (57.6%)	102	487	2.40	4.16	miR159e-5p	Predominance of miR159e-5p within the stringently filtered subset

The numbers of genes with predicted BSs, miRNAs, and total BSs refer to the complete prediction datasets before application of the ΔG ≤ −106 kJ/mol filtering criterion. The principal candidate miRNAs refer to the stringently filtered subset selected for detailed analysis. BS, binding site; TF, transcription factor. Normalized values were calculated from the complete prediction datasets before application of the ΔG ≤ −106 kJ/mol prioritization criterion.

The proportions of genes containing at least one predicted miRNA BS differed significantly between the two species. For NAC genes, the targeted proportion was 81.2% in *O. sativa* (138/170) compared with 46.0% in *Z. mays* (87/189), corresponding to an absolute difference of 35.1 percentage points and an odds ratio of 5.06 (two-sided Fisher’s exact test, P = 3.51 × 10^-12^). For MYB genes, the corresponding proportions were 84.6% (110/130) and 57.6% (117/203), respectively, representing an absolute difference of 27.0 percentage points and an odds ratio of 4.04 (P = 1.61 × 10^-7^). These statistical comparisons quantify differences between the prediction datasets and should not be interpreted as independent biological evidence of species-specific miRNA regulation.

Normalization by gene number further clarified the differences in predicted binding-site abundance. In *O. sativa*, NAC and MYB genes contained 3.48 and 3.69 predicted BSs per analyzed gene, respectively, compared with 1.75 and 2.40 sites per analyzed gene for NAC and MYB genes in *Z. mays*. When calculated only among genes containing at least one predicted site, the corresponding values were 4.28 and 4.36 sites per targeted gene in *O. sativa* and 3.80 and 4.16 in *Z. mays*. Thus, differences based on absolute binding-site totals were not always proportional to differences after normalization, and raw site counts were therefore not interpreted alone as indicators of regulatory complexity.

Rice NAC genes contained the highest absolute number of predicted BSs, whereas rice MYB genes showed the highest proportion of genes containing at least one predicted site; however, these absolute counts were interpreted together with the normalized metrics described above. Within the stringently filtered subset (ΔG ≤ −106 kJ/mol), members of the miR164 family predominated among predicted NAC interactions in both species. Rice MYB transcripts were associated with a more diverse set of candidate miRNAs, particularly miR2102-5p and miR5075-3p, whereas miR159e-5p predominated among the selected maize MYB candidates. Detailed gene-level thermodynamic and positional parameters are presented in the subsequent sections and tables.

### 3.1 Predicted miRNA targeting of NAC and MYB transcription factor genes in *O. sativa*

#### 3.1.1 miRNA BSs in NAC transcription factor genes *in O. sativa*

Among the 170 rice NAC genes analyzed, 138 transcripts contained 591 predicted BSs involving 181 miRNAs, corresponding to 536 unique miRNA–mRNA pairs (**Supplementary Table 1**). After application of the ΔG ≤ −106 kJ/mol criterion, 43 candidate BSs involving 10 miRNAs were retained across 27 NAC genes (**Table 2**). Members of the miR164 family represented the predominant candidate group and were associated with several NAC transcripts, including LOC_Os06g46270.1, LOC_Os12g41680.1, LOC_Os04g38720.1, LOC_Os02g36880.1/4, and LOC_Os06g23650.1. Several of these transcripts were predicted to be compatible with four or five closely related miR164-5p family members. Because these family members may recognize the same or highly overlapping NAC target region, these predictions were interpreted as family-level compatibility rather than as independent miRNA BSs. Outside the miR164 family, miR5075-3p showed the broadest target distribution, whereas miR1848-5p, miR2102-3p/5p, miR1437a/b-3p, miR2927-5p, miR5493-3p, and miR5792-5p showed more restricted candidate-target patterns. Detailed thermodynamic parameters and binding-site positions are provided in **Table 2**, and the comprehensive predicted miR164–NAC pairing schemes are provided in **Supplementary Figure 1**.

**Table 2:** miRNA BSs in NAC transcription factor genes of *O. sativa*.

miRNA	Gene	Start of site, nt	ΔG, kJ/mol	ΔG/ΔGm, %	Length
miR1437a-3p	*LOC_Os03g21030.1*	942	− 108	85	22
miR1437b-3p	*LOC_Os04g42940.1*	33	− 115	82	24
miR164-5p (a, b, d, f)	*LOC_Os06g46270.1*	668 (4)	−106 to −110	91–94	21
	*LOC_Os12g41680.1*	692 (4)	−106 to −110	91–94	21
miR164-5p (a, b, d, e, f)	*LOC_Os04g38720.1*	686 (5)	−106 to −108	91–94	21
	*LOC_Os02g36880.4*	773 (5)	−106 to −108	91–93	21
	*LOC_Os02g36880.1*	776 (5)	−106 to −108	91–93	21
miR164-5p (a, b, d, f)	*LOC_Os06g23650.1*	794 (4)	−108 to −112	83–96	21
miR1848-5p	*LOC_Os05g37080.1*	355	-108	85	21

**Figure 3:**
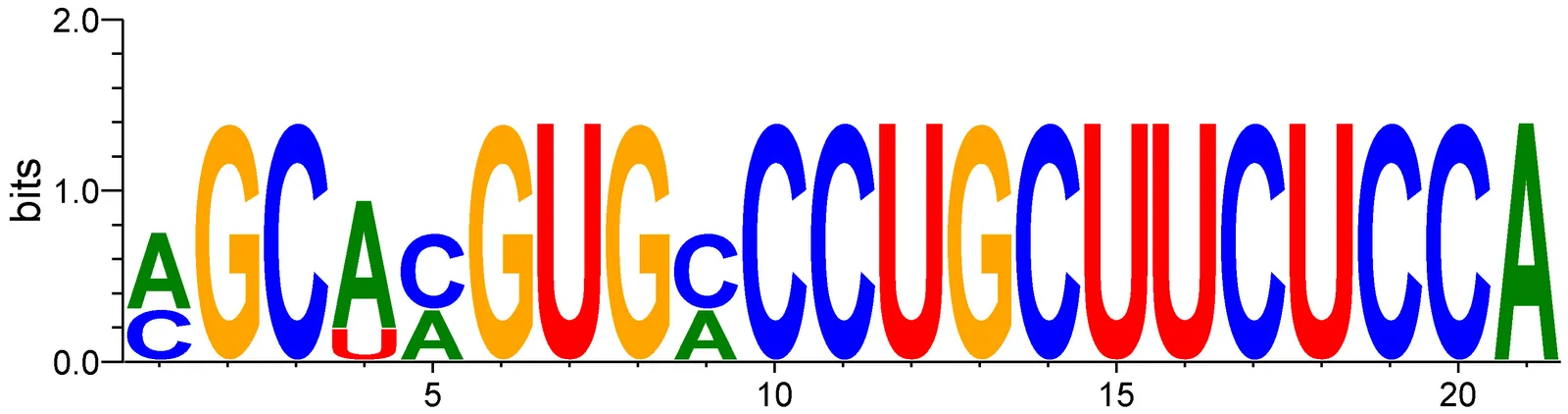
Descriptive sequence logo of selected predicted miR164-compatible sites in NAC transcripts of *O. sativa*. The logo illustrates nucleotide frequencies among the selected sites and does not provide independent evidence of target validity or evolutionary conservation.

Sequence-logo analysis descriptively visualized recurrent nucleotide patterns among the selected predicted miR164-compatible sites, including GC-rich positions in the aligned sequences (**Figure 3**).

Publicly available transcriptomic data confirmed that the selected rice NAC transcripts were expressed across vegetative and reproductive tissues (**Supplementary Figure 2**). Because matched miRNA expression data from the same samples were not available, this analysis was used only to confirm transcript expression and not as evidence of miRNA-mediated regulation.

#### 3.1.2 miRNA BSs in MYB transcription factor genes in *O. sativa*

Among the 130 rice MYB genes analyzed, 110 transcripts contained 480 predicted BSs involving 164 miRNAs (**Supplementary Table 2**). After application of the ΔG ≤ −106 kJ/mol criterion, 11 miRNAs were associated with 20 MYB genes (**Table 3**). miR2102-5p and miR5075-3p showed the broadest candidate-target distributions, each being associated with four MYB transcripts. In contrast, miR1848-5p, miR2104-3p, and miR5819-5p were associated with two genes each, whereas miR159c-3p, miR1437b-3p, miR5792-5p, miR5805-5p, and miR531a/c-5p showed gene-specific prediction patterns.

**Table 3:** miRNA BSs in MYB transcription factor genes of *O. sativa*.

miRNA	Gene	Start of site, nt	ΔG, kJ/mol	ΔG/ΔGm, %	Length
miR1437b-3p	*LOC_Os03g38210.1*	1344	-121	86	24
	*LOC_Os01g63680.1*	846	-115	82	24
miR159c-3p	*LOC_Os05g41166.1*	809	-106	96	21
miR1848-5p	*LOC_Os07g44090.3*	412	-106	83	21
	*LOC_Os07g48870.1*	727	-106	83	21
miR2102-5p	*LOC_Os01g64360.1*	269	-110	91	20
	*LOC_Os05g48010.1*	565	-108	89	20
	*LOC_Os01g62410.1*	98	-106	88	20
	*LOC_Os03g19120.1*	713	-106	88	20
miR2104-3p	*LOC_Os01g16810.1*	505	-108	84	22
	*LOC_Os02g09480.1*	661	-106	82	22
miR5075-3p	*LOC_Os04g38740.1*	403	-113	91	21
	*LOC_Os08g34960.1*	7	-108	88	21
	*LOC_Os04g46384.1*	49	-106	86	21
	*LOC_Os05g04210.1*	747	-106	86	21
miR531a-5p	*LOC_Os08g15020.1*	815	-115	81	24
miR531c-5p	*LOC_Os08g15020.1*	815	-115	81	24
miR5792-5p	*LOC_Os10g33810.1*	504	-108	82	24
miR5805-5p	*LOC_Os05g37730.1*	774	-106	81	24
miR5819-5p	*LOC_Os04g39470.1*	696	-106	86	21
	*LOC_Os06g40330.1*	479	-106	86	21

Comprehensive predicted pairing schemes for the selected rice MYB candidates are provided in **Supplementary Figure 3**, including multi-target candidates miR2102-5p and miR5075-3p and the transcript-specific miR159c-3p–LOC_Os05g41166.1 interaction. The latter showed extensive sequence complementarity and was therefore retained as a candidate for experimental validation. Detailed ΔG, ΔG/ΔGm, binding-site position, and interaction-length values are presented in **Table 3** and are not repeated in the text. Independent psRNATarget analysis subsequently recovered this interaction within a closely corresponding target region, providing additional computational support for its prioritization (Section 3.1.4; **Table 4**).

**Table 4:** Independent computational cross-validation of selected high-priority *O. sativa* miRNA–MYB interactions using psRNATarget.

miRNA	Target transcript	MirTarget site, nt	ΔG, kJ/mol	ΔG/ΔGm, %	psRNATarget site, nt	Expectation	Predicted inhibition	Concordance
osa-miR5075-3p	LOC_Os04g38740.1	403	−113	91	403–423	2.5	Cleavage	Yes
osa-miR159c-3p	LOC_Os05g41166.1	809	−106	96	810–829	0.5	Cleavage	Yes

psRNATarget additionally identified a second candidate osa-miR5075-3p site in LOC_Os04g38740.1 at positions 600–620 nt with an expectation value of 5.0. The predicted inhibition type represents a computational classification by psRNATarget and does not constitute experimental evidence of transcript cleavage. Concordance indicates recovery of the same miRNA–target pair in a corresponding target region by both computational approaches.

#### 3.1.3 Comparative analysis of miRNA BSs in NAC and MYB transcription factor gene families in *O. sativa*

**Figure 4:**
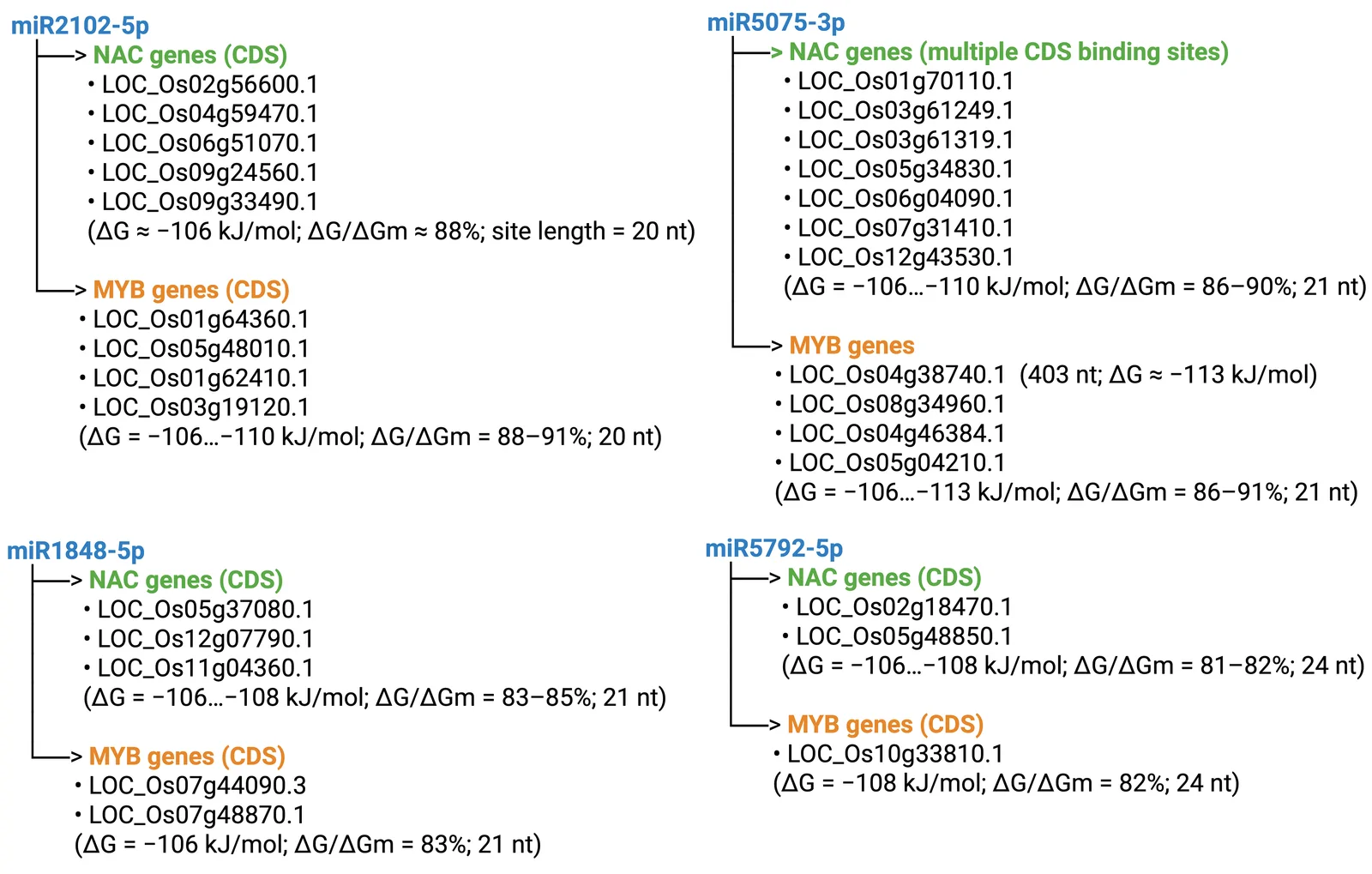
Integrated scheme illustrating shared miRNA targeting patterns of NAC and MYB transcription factor genes in *O. sativa*.

Comparative analysis identified miR2102-5p, miR5075-3p, miR1848-5p, and miR5792-5p as shared candidate miRNAs with predicted BSs in both NAC and MYB transcripts (**Figure 4**). Across the two transcription factor families, the selected interactions showed strongly negative ΔG values and high ΔG/ΔGm ratios, indicating favorable predicted duplex formation. All reported BSs were located within coding regions because only CDS sequences were included in the present analysis. Therefore, this localization reflects the analytical design and should not be interpreted as evidence that miRNA targeting is preferentially restricted to the CDS. Moreover, binding-site position alone cannot distinguish among miRNA-guided transcript cleavage, mRNA destabilization, translational repression, or a non-functional predicted interaction.

Among the shared candidate miRNAs, miR2102-5p showed the broadest distribution across NAC and MYB transcripts. miR5075-3p also formed multiple predicted BSs in both gene families, including transcripts containing more than one site. The predicted miR5075-3p–LOC_Os04g38740.1 interaction at position 403 nt showed near-complete complementarity across the 21-nt binding region and a ΔG value of approximately −113 kJ/mol, identifying this pair as a high-priority candidate for degradome- or reporter-based validation.

Although several other predicted interactions showed more negative absolute ΔG values, miR5075-3p – LOC_Os04g38740.1 was prioritized based on the combined evidence of a favorable ΔG value, a high ΔG/ΔGm ratio of 91%, near-complete complementarity across a canonical 21-nt interaction region, and the broader predicted targeting of both NAC and MYB transcripts by miR5075-3p. In addition, no direct experimental evidence was identified for this specific miRNA–MYB interaction, making LOC_Os04g38740.1 a suitable novel candidate for targeted functional investigation. Its computational prioritization was further supported by psRNATarget, which independently recovered a candidate site spanning positions 403–423 nt, directly corresponding to the principal MirTarget site beginning at position 403 nt (Section 3.1.4; **Table 4**).

In contrast, miR1848-5p and miR5792-5p showed narrower candidate-target distributions across the two transcription factor families. At the family level, NAC transcripts were predominantly associated with members of the miR164 family, whereas MYB transcripts were associated with a more diverse set of candidate miRNAs. The shared miRNAs identified here may therefore represent potential connection points between the predicted NAC and MYB interaction networks. However, the existence of an integrated functional regulatory circuit cannot be established from sequence prediction alone and requires validation using matched miRNA–mRNA expression data, degradome sequencing, or reporter assays.

#### 3.1.4 Independent computational cross-validation of selected miRNA–MYB interactions

To provide independent computational support for selected high-priority MirTarget predictions, two *O. sativa* miRNA–MYB candidate interactions were re-evaluated using psRNATarget. Both selected interactions were independently recovered by the second prediction approach (**Table 4**). For osa-miR5075-3p–LOC_Os04g38740.1, MirTarget identified the prioritized BS beginning at position 403 nt, with a ΔG value of approximately −113 kJ/mol and a ΔG/ΔGm ratio of 91%. psRNATarget independently identified a candidate site spanning positions 403–423 nt with an expectation value of 2.5, showing direct positional concordance with the principal MirTarget prediction. psRNATarget also identified an additional candidate site at positions 600–620 nt with an expectation value of 5.0. For osa-miR159c-3p–LOC_Os05g41166.1, MirTarget identified a candidate BS beginning at position 809 nt, with a ΔG value of −106 kJ/mol and a ΔG/ΔGm ratio of 96%. psRNATarget independently predicted a site spanning positions 810–829 nt with an expectation value of 0.5, corresponding closely to the MirTarget-predicted target region. Thus, both selected high-priority interactions were concordantly predicted by two independent computational approaches. psRNATarget classified the predicted inhibition type as cleavage for both interactions; however, this classification represents a computational prediction and should not be interpreted as experimental evidence of transcript cleavage.

### 3.2 Predicted miRNA targeting of NAC and MYB transcription factor genes in *Z. mays*

#### 3.2.1 miRNA BSs in NAC transcription factor genes in *Z. mays*

A genome-wide *in silico* analysis of miRNA–mRNA interactions in *Z. mays* identified 106 miRNAs with predicted BSs in 87 NAC transcripts, resulting in 331 BSs (**Supplementary Table 3**). Across the complete prediction dataset, ΔG values ranged from −81 to −113 kJ/mol, ΔG/ΔGm ratios ranged from 80% to 96%, and binding-site lengths ranged from 18 to 24 nt. The larger number of BSs relative to targeted transcripts indicates that several NAC transcripts contained multiple predicted miRNA BSs.

After application of the ΔG ≤ −106 kJ/mol criterion, 42 candidate BSs involving nine miRNAs were retained across 11 NAC genes (**Table 5**). Most of these candidates involved members of the miR164-5p family, including miR164a-5p, miR164b-5p, miR164c-5p, miR164d-5p, miR164e-5p, miR164f-5p, miR164g-5p, and miR164h-5p, whereas miR167d-3p represented an additional candidate miRNA.

**Table 5:** miRNA BSs in NAC transcription factor genes of *Z. mays*.

miRNA	Gene	Start of site, nt	ΔG, kJ/mol	ΔG/ΔGm, %	Length
miR164h-5p	*GRMZM2G009892_P01*	692	-108	93	21
	*GRMZM2G009892_P02*				
	*GRMZM2G009892_P03*				
miR164-5p (a, b, c, d, f, g)	*GRMZM2G063522_P01*	659 (6)	−106 to −110	91–95	21
	*GRMZM2G114850_P01*	698 (6)	−106 to −110	91–95	21
	*GRMZM2G139700_P01*	737 (6)	−108 to −113	93–96	21
miR164-5p (e, h)	*GRMZM2G146380_P01*	434 (2)	−106 to −110	93–95	21
miR164-5p (a, b, c, d, f, g)	*GRMZM2G393433_P01*	755 (6)	−108 to −113	93–96	21
	*GRMZM2G393433_P02*	758 (6)	−108 to −113	93–96	21
miR164-5p (e, h)	*GRMZM5G898290_P01*	722	−106 to −110	93–95	21
	*GRMZM5G898290_P02*				

Values in parentheses () denote the number of BSs.

Multiple closely related miR164-5p family members were predicted to be compatible with NAC transcripts such as GRMZM2G063522, GRMZM2G114850, GRMZM2G139700, and GRMZM2G393433. Because closely related miR164-5p sequences may recognize the same or highly overlapping NAC target region, these predictions were interpreted as a recurrent family-level compatibility pattern rather than as evidence of multiple independent regulatory inputs. Comprehensive predicted miRNA–NAC pairing schemes are provided in **Supplementary Figure 4**.

**Figure 5:**
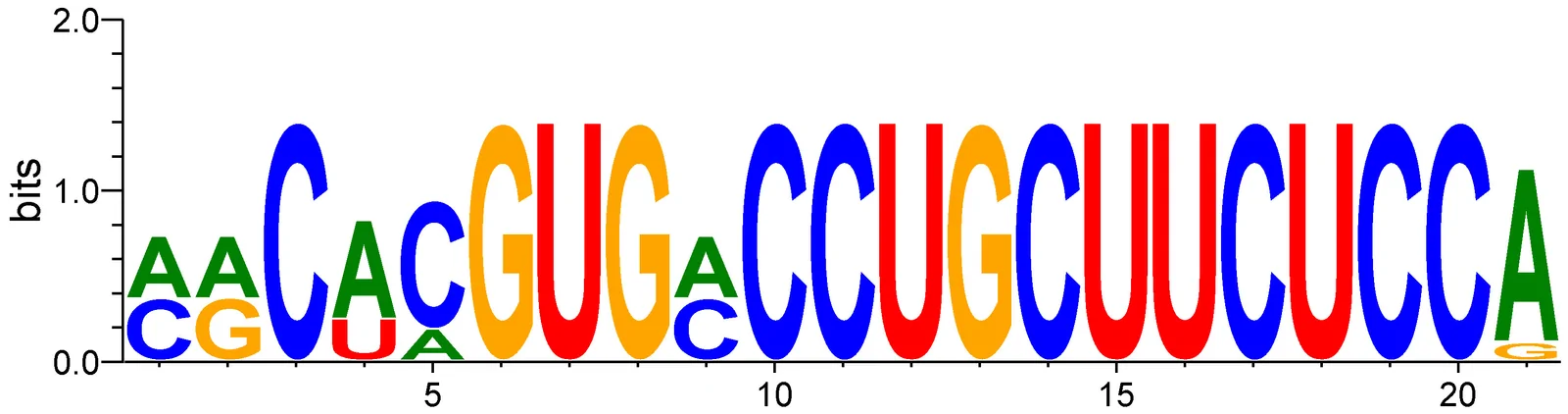
Descriptive sequence logo of selected predicted miR164-compatible sites in NAC transcripts of *Z. mays*. The logo illustrates nucleotide frequencies among the selected sites and does not provide independent evidence of target validity or evolutionary conservation.

The sequence logo descriptively illustrated recurrent nucleotide patterns among the selected predicted miR164-compatible sites, including GC-rich positions in the aligned regions (**Figure 5**). All reported sites were located within coding regions because only CDS sequences were analyzed. These sequence and thermodynamic characteristics support the prioritization of the predicted miR164–NAC duplexes as candidates for experimental validation but do not independently demonstrate coordinated post-transcriptional regulation.

#### 3.2.2 miRNA BSs in MYB transcription factor genes in *Z. mays*

Analysis of miRNA–mRNA interactions in *Z. mays* identified 487 predicted BSs involving 102 miRNAs across 117 of the 203 MYB genes examined (**Supplementary Table 4**). In the complete prediction dataset, ΔG values ranged from −81 to −115 kJ/mol, ΔG/ΔGm ratios ranged from 80% to 100%, and binding-site lengths ranged from 18 to 22 nt. These values describe the complete prediction dataset and should not be interpreted as a high-confidence threshold.

Within the subset selected using the ΔG ≤ −106 kJ/mol criterion, miR159e-5p was the predominant candidate miRNA. It formed multiple predicted BSs in MYB transcripts, including AC217264.3_FGP005 and GRMZM2G070523. The recurrent occurrence of miR159e-5p sites suggests that this miRNA may represent an important candidate for post-transcriptional regulation of maize MYB transcripts.

Comprehensive predicted miR159e-5p–MYB pairing schemes are provided in **Supplementary Figure 5**. These characteristics support prioritization of the selected interactions for experimental validation but do not independently demonstrate functional miRNA–mediated regulation.

#### 3.2.3 Comparative analysis of miRNA BSs in NAC and MYB transcription factor gene families in *Z. mays*

MYB transcripts contained 487 predicted miRNA BSs, compared with 331 sites in NAC transcripts. After normalization to the complete analyzed gene sets, this corresponded to 2.40 sites per analyzed MYB gene and 1.75 sites per analyzed NAC gene. Among targeted genes, the corresponding values were more similar, at 4.16 sites per MYB gene and 3.80 sites per NAC gene. Thus, the higher absolute site count in the MYB dataset was accompanied by a higher normalized abundance per analyzed gene, whereas the difference among targeted genes was comparatively modest.

Within the stringently filtered subsets, NAC transcripts were predominantly associated with members of the miR164-5p family, which formed multiple predicted sites across several NAC transcripts. In contrast, miR159e-5p was the predominant candidate miRNA among the selected MYB interactions and was associated with multiple MYB transcripts.

Both NAC and MYB transcripts frequently contained more than one predicted miRNA BSs indicating recurrent multiple-site targeting patterns. The two transcription factor families therefore differed mainly in their predominant candidate miRNA families and in the distribution of predicted sites. These differences suggest possible functional specialization but do not establish distinct regulatory mechanisms without experimental validation.

## 4 Discussion

NAC and MYB transcription factor genes are central regulators of cellulose biosynthesis and SCW formation in higher plants (Nakano et al., 2015; Xiao et al., 2021). In cereals such as rice and maize, coordinated NAC–MYB activity is essential for proper SCW deposition, stem mechanical strength, and biomass accumulation, underscoring their pivotal role in determining cell wall architecture and cellulose content (Zhong et al., 2011).

As demonstrated by Yoshida et al. (2013), rice secondary wall NAC domain proteins (OsSWNs) play a key role in regulating SCW thickening and cellulose-related traits (Yoshida et al., 2013). Our analysis predicted numerous miRNA BSs in NAC transcripts, with members of the miR164 family predominating among the thermodynamically prioritized candidates. Several NAC transcripts contained multiple thermodynamically favorable predicted sites, suggesting a potential candidate-targeting pattern that requires experimental validation. The presence of several highly stable miRNA BSs (ΔG ≤ −106 kJ/mol) within individual NAC transcripts suggests that miRNA–mediated post-transcriptional regulation may act as a fine-tuning mechanism modulating the activity of OsSWN-related regulatory pathways during SCW formation.

The ΔG ≤ −106 kJ/mol threshold was used to select a stringent subset of thermodynamically favorable predicted miRNA–mRNA duplexes for detailed comparative analysis. This operational cut-off facilitated the consistent filtering of the rice and maize datasets but does not represent a universally established biological threshold. Strongly negative ΔG values and high ΔG/ΔGm ratios increase confidence in predicted duplex formation; however, they do not independently demonstrate that an interaction occurs *in vivo* or results in transcript cleavage, mRNA destabilization, or translational repression. Therefore, experimental validation is required to determine the functional relevance of the selected candidate interactions. Previous studies, including our own, have used strongly negative free energy values as one criterion for prioritizing thermodynamically favorable candidate miRNA–mRNA interactions (Bari et al., 2013; Ivashchenko et al., 2016b). In the present dataset, CDS localization reflects the use of CDS-only input sequences and therefore cannot be used to infer a specific regulatory mechanism. Although CDS-localized miRNA interactions have been associated with translational repression or mRNA destabilization in previous studies (Hausser et al., 2013; Brümmer and Hausser, 2014), such mechanisms cannot be inferred from localization alone in the present analysis. The ΔG cutoff was used only as an operational criterion for prioritizing thermodynamically favorable predicted interactions.

Additional evidence for the conserved role of miR164 in SCW regulation is provided by the genome-wide study of NAC transcription factors in moso bamboo by Shan et al. (2019), where miR164 target sites were identified in multiple SCW-associated PeNAC genes showing inverse expression patterns relative to miR164 (Shan et al., 2019). This observation is consistent with our finding that miR164-family members predominated among the thermodynamically prioritized NAC candidate interactions.

More direct experimental evidence is available for several rice miR164–NAC interactions identified in the present analysis. Fang et al. (2014) identified six miR164-targeted NAC genes in rice, designated OMTN1–OMTN6, corresponding to LOC_Os02g36880, LOC_Os04g38720, LOC_Os12g41680, LOC_Os06g46270, LOC_Os06g23650, and LOC_Os08g10080. Notably, five of these genes were also recovered among the thermodynamically prioritized candidate targets in our analysis. RLM-5′ RACE directly detected miR164-guided cleavage products for OMTN1/LOC_Os02g36880 and OMTN2/LOC_Os04g38720, providing transcript-specific experimental support for these two predicted interactions. The remaining miR164–NAC pairs identified here should nevertheless be considered candidate interactions unless comparable cleavage or reporter-based evidence is available (Fang et al., 2014).

Experimental evidence also supports the miR164–NAC module in maize. Li et al. (2012) demonstrated that maize miR164 directs cleavage of endogenous ZmNAC1 mRNA and reported opposite expression patterns of miR164 and ZmNAC1 in two maize inbred lines. ZmNAC1 corresponds to GRMZM2G063522, which was also identified as a thermodynamically prioritized candidate target in the present study. The previously reported cleavage and expression evidence therefore provides independent experimental support for this specific maize interaction. However, the other predicted maize miR164–NAC pairs still require transcript-specific validation (Li et al., 2012).

**Figure 6:**
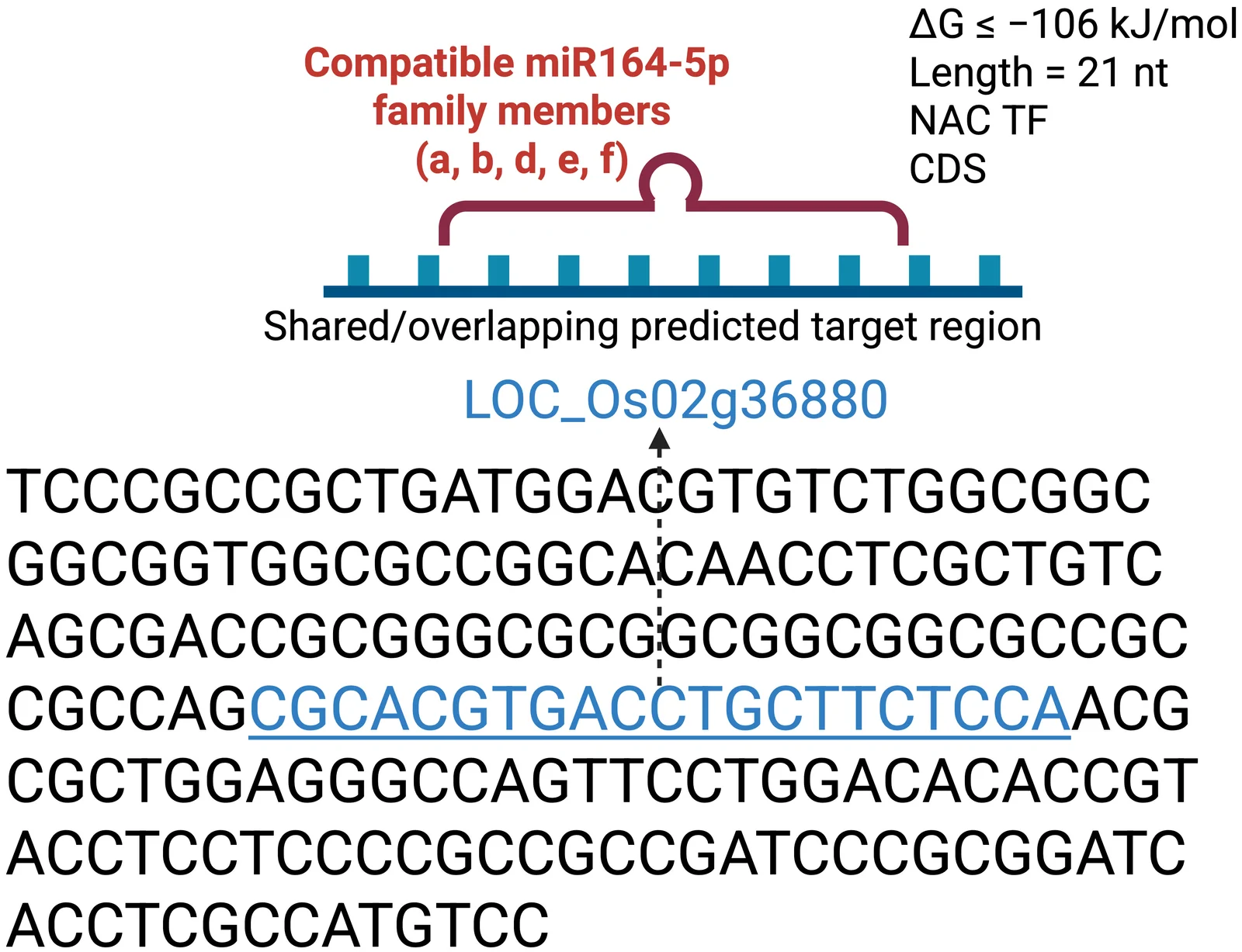
Predicted compatibility of closely related miR164-5p family members with a shared target region within the analyzed CDS sequence of the NAC transcript LOC_Os02g36880 of *O. sativa*.

Notably, several NAC transcripts were predicted to be compatible with multiple closely related members of the miR164-5p family (**Figure 6**). In *O. sativa*, miR164a/b/d/e/f-5p variants were predicted to recognize the same or highly overlapping regions within the analyzed CDS sequences in NAC transcripts such as LOC_Os02g36880 and LOC_Os04g38720. Similar family-level prediction patterns were observed in *Z. mays*. Because closely related miR164 family members have highly similar sequences, their compatibility with a single shared target region should not be interpreted as multiple independent miRNA BSs or independent regulatory inputs. The present computational analysis therefore supports family-level target compatibility but does not establish redundancy, cooperativity, competition, or simultaneous regulation by different miR164 family members. These possible regulatory relationships remain hypotheses requiring experimental validation.

**Figure 7:**
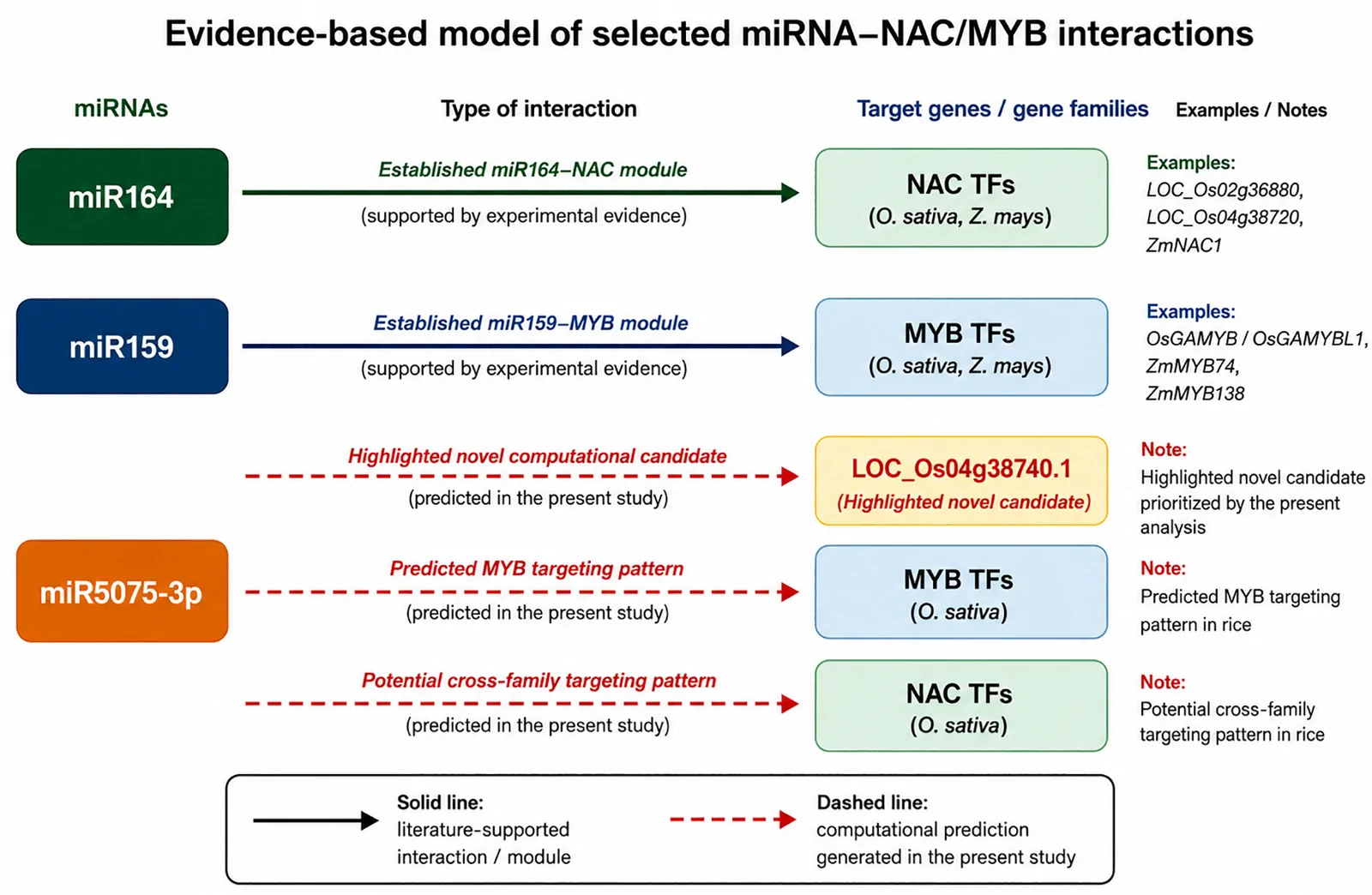
Evidence-based model of selected miRNA–NAC/MYB interactions.

In contrast to NAC transcripts, MYB transcripts were associated with a more diverse set of predicted miRNAs, suggesting possible functional specialization of post-transcriptional targeting within the SCW regulatory network. MYB transcription factors typically act downstream of NAC proteins and directly modulate the expression of cellulose synthase (CesA) genes and other structural components of the cell wall. The broader miRNA repertoire targeting MYB genes observed in this study may reflect the need for fine spatial and temporal regulation of downstream transcriptional outputs during SCW deposition. Experimental evidence supports the biological plausibility of the miR159–MYB regulatory module in cereals. In rice, 5′-RACE analysis detected miR159-guided cleavage products for OsGAMYB and OsGAMYBL1 in both vegetative and floral tissues, while inhibition of miR159 in MIM159 transgenic plants increased the transcript abundance of these target genes (Zheng et al., 2020). In maize, degradome analysis and RLM-5′ RACE identified cleavage of ZmMYB74 and ZmMYB138 by zma-miR159, and dual-luciferase assays further confirmed direct targeting. Transgenic overexpression of zma-miR159 and CRISPR/Cas9-mediated disruption of ZmMYB74 also supported the functional importance of this module in maize grain development (Wang et al., 2023). These studies provide strong experimental support for the broader cereal miR159–MYB module. However, they do not directly validate the specific miR159c-3p–LOC_Os05g41166.1 interaction in rice or the AC217264.3_FGP005 and GRMZM2G070523 interactions predicted in maize. Accordingly, despite independent computational support from psRNATarget, the miR159c-3p–LOC_Os05g41166.1 pair remains classified as a computational candidate because the available experimental evidence concerns other members of the broader miR159–MYB regulatory module. These transcript-specific pairs should therefore remain classified as computational candidates requiring direct validation. The identification of candidate miRNAs with predicted BSs in both NAC and MYB transcripts suggests possible connection points between these interaction networks. Shared candidates such as miR5075-3p may potentially contribute to coordinated post-transcriptional regulation; however, this hypothesis requires experimental validation. The principal literature-supported and computationally predicted miRNA–NAC/MYB interactions are summarized in **Figure 7**, where solid lines indicate relationships supported by published experimental evidence and dashed lines indicate candidate interactions predicted in the present study. The principal candidate miRNAs also differ in the strength of their annotation and experimental support in miRBase. Therefore, less extensively characterized candidates, particularly miR5075-3p, miR2102-5p, and miR159e-5p, were interpreted cautiously and were not used alone to support the main biological conclusions.

Previous computational studies have identified miR5075-3p as a potential multi-target miRNA associated with several transcription factor families (Rakhmetullina et al., 2021). Independent degradome analysis in rice identified multiple miR5075-associated target transcripts, and subsequent expression and functional analyses supported the biological relevance of the miR5075–Os02g0817500 relationship in rice seed vigor (Zhou et al., 2022). However, Os02g0817500 is distinct from the MYB transcripts prioritized in the present study. Therefore, this evidence supports the broader biological activity of miR5075 in rice but does not provide transcript-specific experimental validation for miR5075-3p–LOC_Os04g38740.1 or the other miR5075-3p–MYB candidate interactions listed in **Table 6**.

**Table 6:** Experimental evidence status of selected key miRNA–target interactions discussed in the present study.

Interaction	Degradome /PARE	RLM-5′ RACE / 5′-RACE	Reporter assay	Genetic/functional evidence	Expression evidence	Reference
*O. sativa* miR164–LOC_Os02g36880 (*OMTN1*)	–	Yes	–	–	–	Fang et al., 2014
*O. sativa* miR164–LOC_Os04g38720 (*OMTN2*)	–	Yes	–	Yes (*OMTN2* overexpression)	–	Fang et al., 2014
*Z. mays* miR164–GRMZM2G063522 (*ZmNAC1*)	–	Yes	–	Yes (*ZmNAC1* overexpression)	Yes, inverse miR164/*ZmNAC1* expression	Li et al., 2012
*O. sativa* miR159–*OsGAMYB*/*OsGAMYBL1*	–	Yes	–	Yes, MIM159 transgenic rice	Yes	Zheng et al., 2020
*Z. mays* miR159–*ZmMYB74*	Yes	Yes	Yes	Yes, miR159 OE + *ZmMYB74* CRISPR/Cas9	–	Wang et al., 2023
*Z. mays* miR159–*ZmMYB138*	Yes	Yes	Yes	–	–	Wang et al., 2023
*O. sativa* miR159c-3p–LOC_Os05g41166.1	–	–	–	–	–	Present study
*O. sativa* miR5075-3p–LOC_Os04g38740.1	–	–	–	–	–	Present study

“–” indicates that no direct evidence was identified. The miR159c-3p–LOC_Os05g41166.1 and miR5075-3p–LOC_Os04g38740.1 interactions are supported computationally by MirTarget and psRNATarget but remain experimentally unvalidated.

To systematically distinguish the levels of evidence, the selected miRNA–target relationships were classified according to validation type (**Table 6**). The findings were interpreted as: (i) established miR164–NAC and miR159–MYB interactions recovered as positive benchmarks; (ii) candidates with indirect prior support for the same miRNA or regulatory module but not the same transcript pair; and (iii) novel transcript-specific computational candidates, particularly miR5075-3p–LOC_Os04g38740.1, requiring direct experimental validation. Evidence involving a different target transcript was not considered direct validation of a predicted pair.

Among these novel candidates, the miR5075-3p–LOC_Os04g38740.1 interaction was prioritized as a leading testable computational hypothesis for direct experimental validation. This prioritization was based on the combined evidence of a favorable ΔG value of approximately −113 kJ/mol, a high ΔG/ΔGm ratio of 91%, near-complete complementarity across the 21-nt predicted binding region, and the predicted targeting of transcripts from both the NAC and MYB transcription factor families by miR5075-3p. However, this prioritization does not imply that miR5075-3p necessarily binds first or exclusively *in vivo*. More generally, when a transcript contains BSs for multiple candidate miRNAs, its regulation may be combinatorial. Overlapping or closely positioned sites may restrict the simultaneous occupancy of different AGO–miRNA complexes and potentially result in competition, whereas sufficiently separated sites may permit independent or cooperative regulatory effects. These possibilities represent mechanistic hypotheses only and cannot be inferred from target-prediction results without experimental evidence of site occupancy and regulatory effects. Previous studies have demonstrated that binding-site arrangement can influence miRNA-mediated repression and that, in plants, silencing efficiency also depends on miRNA–target complementarity and the local secondary structure of the target RNA (Hon and Zhang, 2007; Liu et al., 2014; Zheng et al., 2017). Therefore, the most negative ΔG value should be regarded as one prioritization parameter rather than evidence that a particular miRNA binds first or excludes other candidate miRNAs. The proposed miR5075-3p–LOC_Os04g38740.1 interaction should be examined using degradome sequencing or RLM-5′ RACE, reporter assays, matched miRNA–mRNA expression analysis, and site-specific mutagenesis to establish its functional relevance.

Independent computational cross-validation further supported the prioritization of the two selected rice MYB candidate interactions. The osa-miR5075-3p–LOC_Os04g38740.1 interaction was independently recovered by psRNATarget at positions 403–423 nt with an expectation value of 2.5, directly corresponding to the principal MirTarget site beginning at position 403 nt. Similarly, osa-miR159c-3p–LOC_Os05g41166.1 was independently predicted at positions 810–829 nt with an expectation value of 0.5, closely corresponding to the MirTarget site beginning at position 809 nt. Concordance between the two prediction approaches provides additional computational support for prioritizing these specific candidate interactions. Nevertheless, agreement between computational algorithms does not establish an interaction *in vivo* or demonstrate functional regulation, and direct experimental validation remains necessary.

Comparative analysis identified similar predicted miR164–NAC interaction patterns in *O. sativa* and *Z. mays*. These cross-species similarities are consistent with the hypothesis of possible evolutionary conservation. However, similarity in sequence complementarity, CDS localization, and thermodynamic parameters alone does not establish conservation of functional regulatory mechanisms. The observed differences between rice and maize should therefore be interpreted as candidate species-associated patterns requiring comparative expression and functional validation. The statistical differences in targeted-gene proportions should be interpreted within the same computational context. Although Fisher’s exact tests identified marked differences between the rice and maize prediction datasets, the analyzed genes and predicted BSs do not represent independent biological replicates. Therefore, the reported P values and effect sizes quantify differences in predicted targeting patterns rather than demonstrating species-level differences in miRNA regulatory activity *in vivo*. Despite providing a systematic cross-species analysis of predicted miRNA targeting within NAC and MYB transcription factor families, this study has certain limitations. Experimental validation of the predicted miRNA–mRNA interactions using degradome sequencing, reporter assays, or transgenic approaches will be necessary to confirm their functional relevance. Future studies integrating matched miRNA and mRNA expression data may clarify whether the predicted interactions contribute to secondary cell wall–related regulatory processes. A further limitation is that only CDS regions were included in the target-prediction analysis. Although this approach ensured consistent transcript coverage and cross-species comparability, it excluded potential miRNA BSs located in the 5′UTR and 3′UTR. Consequently, the number and regional distribution of BSs reported here should not be interpreted as representing the complete miRNA target landscape of NAC and MYB transcripts. Future studies should incorporate full-length, isoform-specific transcripts and evaluate candidate BSs across the 5′UTR, CDS, and 3′UTR using harmonized genome annotations.

The tissue-specific expression analysis provided additional context by confirming that selected rice NAC candidate transcripts are expressed across vegetative and reproductive tissues. However, this analysis was exploratory and was limited to rice NAC genes. Consequently, formal miRNA–target expression correlation analysis was not performed. Combining expression values from different genotypes, tissues, developmental stages, or independent studies could introduce substantial biological and technical confounding. Future studies should therefore integrate matched small RNA-seq and mRNA-seq datasets from identical samples to test for inverse miRNA–target expression relationships, followed by degradome, reporter-based, or genetic validation.

## 5 Conclusion

This study provides a comparative in silico characterization of predicted miRNA targeting across the complete annotated NAC and MYB transcription factor gene sets of *O. sativa* and *Z. mays*. Rice showed higher proportions of NAC and MYB genes containing predicted miRNA BSs than maize, while normalization by gene number confirmed distinct quantitative targeting patterns between the two species and transcription factor families. The analysis recovered the established miR164–NAC and miR159–MYB modules as positive benchmarks, whereas transcript-specific predictions were distinguished from experimentally supported interactions. Among the novel candidates, miR5075-3p–LOC_Os04g38740.1 was prioritized as the leading testable computational hypothesis based on favorable thermodynamic parameters, near-complete complementarity, and concordant MirTarget and psRNATarget predictions. The miR159c-3p–LOC_Os05g41166.1 interaction also received independent computational support. These prioritized candidates provide specific targets for future degradome, reporter-based, and site-specific functional validation.

## Data Availability

The original contributions presented in the study are included in the article/**Supplementary Material**, further inquiries can be directed to the corresponding author/s.
