# Supplementary material for: Comparative in silico analysis of miRNA targeting of NAC and MYB transcription factors associated with secondary cell wall regulation in rice and maize
Source: Front Plant Sci. 2026 Sep 3;17:1887120. doi: 10.3389/fpls.2026.1887120 (PMC13582377; doi:10.3389/fpls.2026.1887120)
Supplement: Supplementary file 1 [file DataSheet1.pdf]

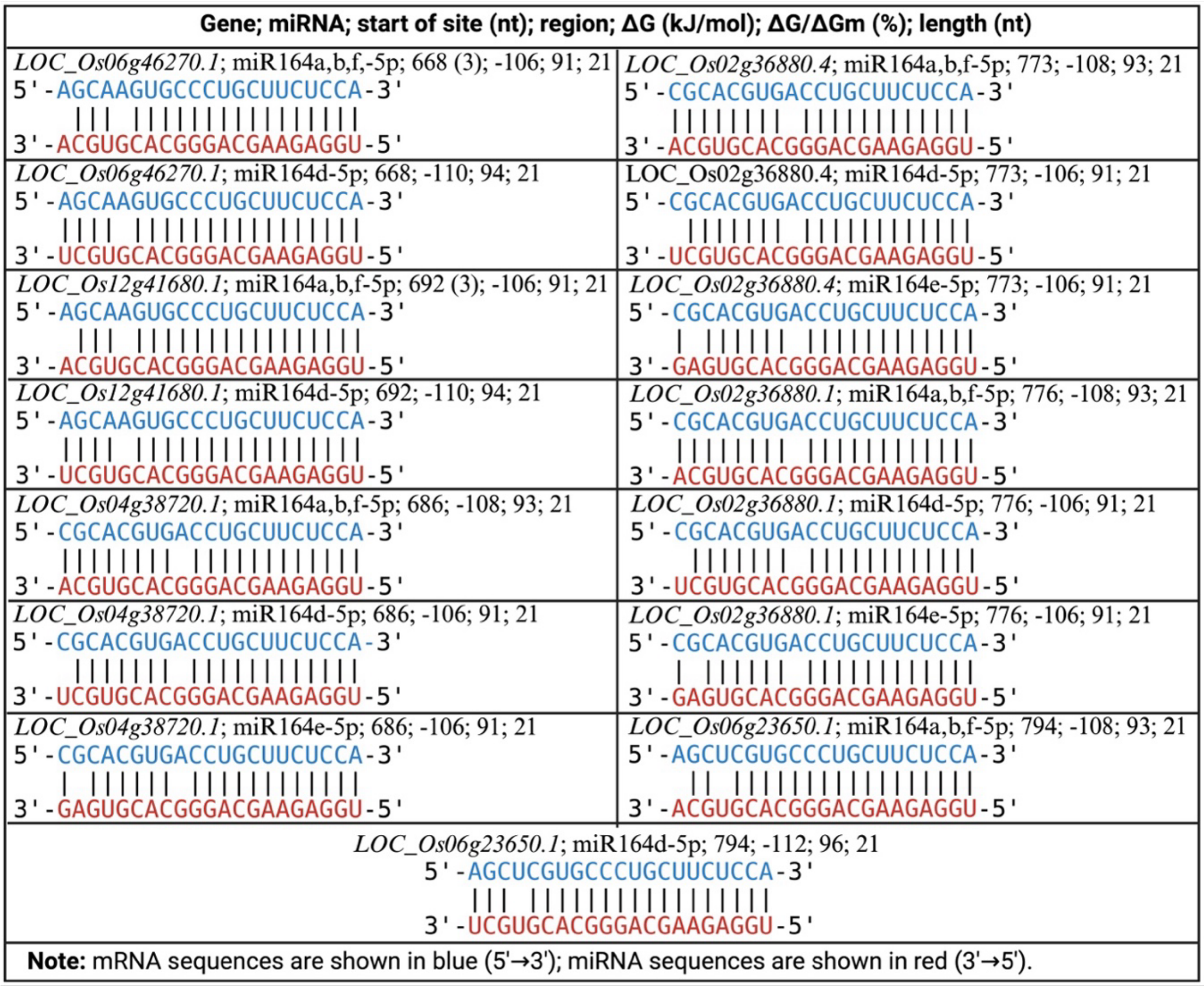

Supplementary Figure S1. Comprehensive predicted base-pairing schemes of miR164-5p family members with NAC transcripts of *O. sativa*. The diagrams show the predicted miRNA–mRNA duplexes retained according to the stringent operational criterion (ΔG ≤ −106 kJ/mol).

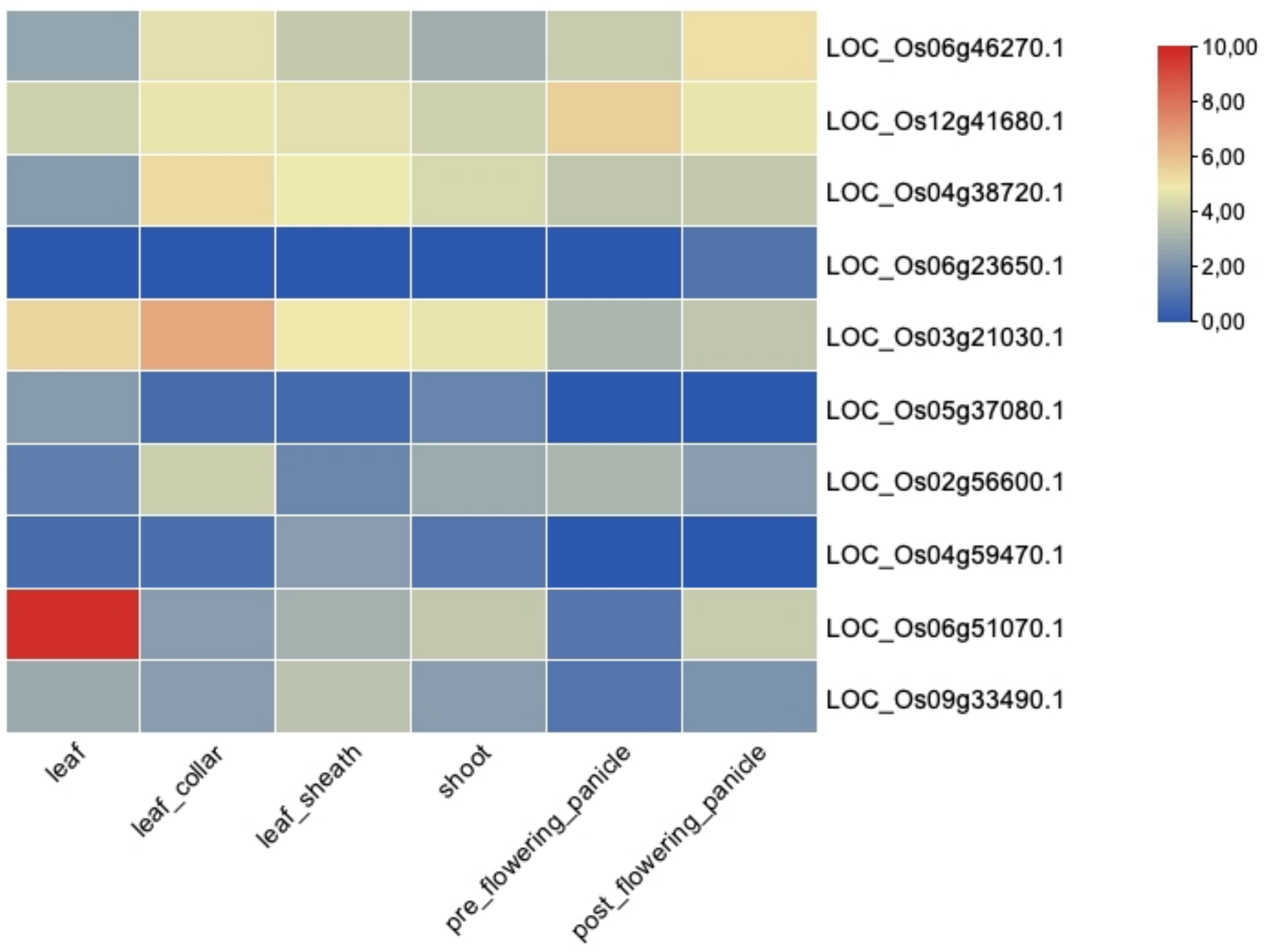

Supplementary Figure S2. Tissue-specific expression profiles of selected rice NAC transcripts. Expression levels (TPM) were obtained from publicly available RNA-seq datasets and visualized across vegetative and reproductive tissues as log2(TPM + 1). The analysis was used only to confirm transcript expression and should not be interpreted as evidence of miRNA-mediated regulation.

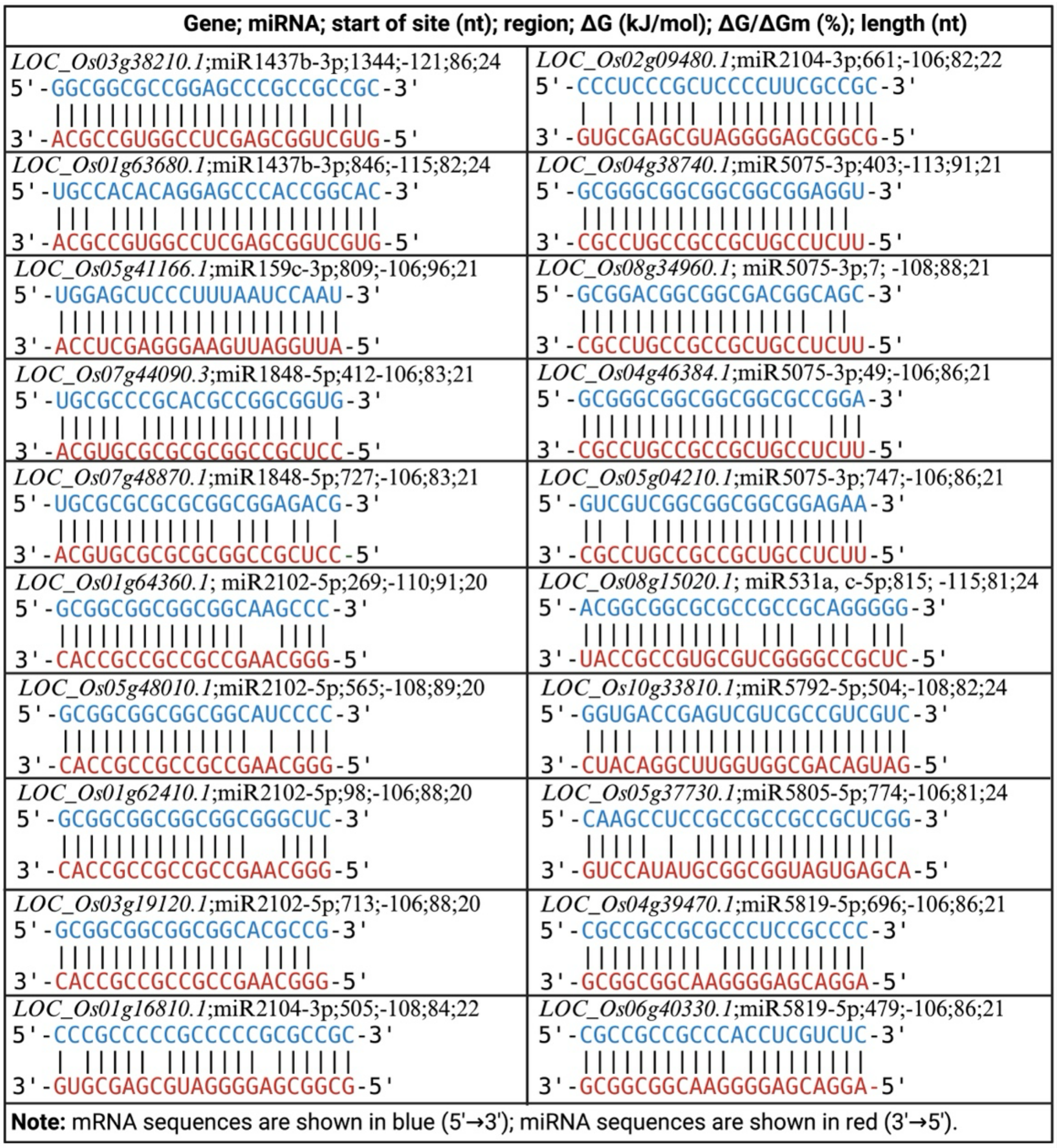

Supplementary Figure S3. Comprehensive predicted base-pairing schemes between selected miRNAs and MYB transcription factor transcripts of *O. sativa*. The diagrams show the predicted miRNA–mRNA duplexes retained according to the stringent operational criterion ($\Delta G \leq -106$ kJ/mol).

<table>
<tr><th colspan="2">Gene; miRNA; start of site (nt); region; ΔG (kJ/mol); ΔG/ΔGm (%); length (nt)</th></tr>
<tr><td>GRMZM2G009892_P01,03;miR164h-5p;692;-108;93;21
5'-CACACGUGACCUGCUUCUCCG-3'
   |||||||| ||||||||||||
3'-GUGUGCACGGGACGAAGAGGU-5'</td><td>GRMZM2G146380_P01;miR164h-5p;434;-110;95;21
5'-CACACGUGACCUGCUUCUCCA-3'
   |||||||| ||||||||||||
3'-GUGUGCACGGGACGAAGAGGU-5'</td></tr>
<tr><td>GRMZM2G063522_P01;miR164a,b,c,d,g-5p;659;-106;91;21
5'-AGCAAGUGCCCUGCUUCUCCA-3'
    ||| ||||||||||||||||
3'-ACGUGCACGGGACGAAGAGGU-5'</td><td>GRMZM2G393433_P01;miR164a,b,c,d,g-5p;755;-108;93
5'-AGCUCGUGCCCUGCUUCUCCA-3'
    || |||||||||||||||||
3'-ACGUGCACGGGACGAAGAGGU-5'</td></tr>
<tr><td>GRMZM2G063522_P01;miR164f-5p;659;-110;95;21
5'-AGCAAGUGCCCUGCUUCUCCA-3'
   |||| ||||||||||||||||
3'-UCGUGCACGGGACGAAGAGGU-5'</td><td>GRMZM2G393433_P01;miR164f-5p;755;-113;96;21
5'-AGCUCGUGCCCUGCUUCUCCA-3'
   ||| |||||||||||||||||
3'-UCGUGCACGGGACGAAGAGGU-5'</td></tr>
<tr><td>GRMZM2G114850_P01;miR164a,b,c,d,g-5p;698;-106;91;21
5'-AGCAGGUGCCCUGCUUCUCCA-3'
    ||| ||||||||||||||||
3'-ACGUGCACGGGACGAAGAGGU-5'</td><td>GRMZM2G393433_P02;miR164a,b,c,d,g-5p;758;-108;93;21
5'-AGCUCGUGCCCUGCUUCUCCA-3'
    || |||||||||||||||||
3'-ACGUGCACGGGACGAAGAGGU-5'</td></tr>
<tr><td>GRMZM2G114850_P01;miR164f-5p;698;-110;95;21
5'-AGCAGGUGCCCUGCUUCUCCA-3'
   |||| ||||||||||||||||
3'-UCGUGCACGGGACGAAGAGGU-5'</td><td>GRMZM2G393433_P02;miR164f-5p;758;-113;96;21
5'-AGCUCGUGCCCUGCUUCUCCA-3'
   ||| |||||||||||||||||
3'-UCGUGCACGGGACGAAGAGGU-5'</td></tr>
<tr><td>GRMZM2G139700_P01;miR164a,b,c,d,g-5p;737;-108;93;21
5'-AGCUCGUGCCCUGCUUCUCCA-3'
    || |||||||||||||||||
3'-ACGUGCACGGGACGAAGAGGU-5'</td><td>GRMZM5G898290_P01;miR164e-5p;722;-106;93;21
5'-CACACGUGACCUGCUUCUCCA-3'
   | |||||| ||||||||||||
3'-GAGUGCACAGGACGAAGAGGU-5'</td></tr>
<tr><td>GRMZM2G139700_P01;miR164f-5p;737;-113;96;21
5'-AGCUCGUGCCCUGCUUCUCCA-3'
   ||| |||||||||||||||||
3'-UCGUGCACGGGACGAAGAGGU-5'</td><td>GRMZM5G898290_P01;miR164h-5p;722;-110;95;21
5'-CACACGUGACCUGCUUCUCCA-3'
   |||||||| ||||||||||||
3'-GUGUGCACGGGACGAAGAGGU-5'</td></tr>
<tr><td>GRMZM2G146380_P01;miR164e-5p;434;-106;93;21
5'-CACACGUGACCUGCUUCUCCA-3'
   | |||||| ||||||||||||
3'-GAGUGCACAGGACGAAGAGGU-5'</td><td>GRMZM5G898290_P02;miR164e-5p;722;-106;93;21
5'-CACACGUGACCUGCUUCUCCA-3'
   | |||||| ||||||||||||
3'-GAGUGCACAGGACGAAGAGGU-5'</td></tr>
<tr><td colspan="2">GRMZM5G898290_P02;miR164h-5p;722;-110;95;21
5'-CACACGUGACCUGCUUCUCCA-3'
   |||||||| ||||||||||||
3'-GUGUGCACGGGACGAAGAGGU-5'</td></tr>
<tr><td colspan="2">Note: mRNA sequences are shown in blue (5'→3'); miRNA sequences are shown in red (3'→5').</td></tr>
</table>

Supplementary Figure S4. Comprehensive predicted base-pairing schemes of miRNAs with NAC transcription factor transcripts of *Z. mays*. The diagrams show the predicted miRNA–mRNA duplexes retained according to the stringent operational criterion (ΔG ≤ −106 kJ/mol).

<table>
<tr><th colspan="2">Gene; miRNA; start of site (nt); region; ΔG (kJ/mol); ΔG/ΔGm (%); length (nt)</th></tr>
<tr><td>AC217264.3_FGP005;miR159e-5p;1009 (2);-115;100;21
5'-GAACAGAUGCUGCAGGAGCUG-3'
   |||||||||||||||||||||
3'-CUUGUCUACGACGUCCUCGAC-5'</td><td>GRMZM2G070523_P01;miR159e-3p;869;-110;100;21
5'-UGGAGCUCCCUUCAAACCAAU-3'
   |||||||||||||||||||||
3'-ACCUCGAGGGAAGUUUGGUUA-5'</td></tr>
<tr><td colspan="2">Note: mRNA sequences are shown in blue (5'→3'); miRNA sequences are shown in red (3'→5').</td></tr>
</table>

Supplementary Figure S5. Comprehensive predicted base-pairing schemes of miRNAs with MYB transcription factor transcripts of *Z. mays*. The diagrams show the predicted miRNA–mRNA duplexes retained according to the stringent operational criterion (ΔG ≤ −106 kJ/mol).
